# Insecticide-impregnated netting as a potential tool for long-lasting control of the leishmaniasis vector *Lutzomyia longipalpis* in animal shelters

**DOI:** 10.1186/1756-3305-6-133

**Published:** 2013-05-04

**Authors:** Daniel Peter Bray, James G C Hamilton

**Affiliations:** 1 Centre for Applied Entomology and Parasitology, Institute for Science and Technology in Medicine, Keele University, Keele, Staffordshire ST5 5BG, UK

**Keywords:** Lambda-cyhalothrin, Leishmaniasis, *Lutzomyia longipalpis*, Permethrin, LLIN

## Abstract

**Background:**

Leishmaniasis remains a serious neglected disease, with more than 350 million people potentially at risk worldwide. Control strategies often rely on spraying residual insecticides to target populations of the sand fly vectors that transmit *Leishmania* parasites when blood-feeding. These programmes are often difficult to sustain effectively, as sand fly resting sites must be resprayed on a regular basis. Here, we investigate whether application of insecticide-impregnated netting to a surface could act as an alternative to residual spraying for controlling the American visceral leishmaniasis vector *Lutzomyia longipalpis*.

**Methods:**

Female *L. longipalpis* from our laboratory colony were exposed for 1 h to three treatments applied to plywood surfaces: 2% permethrin-impregnated netting (Olyset®), 20 mg a.i.m^-2^ micro-encapsulated lambda-cyhalothrin (Demand CS®) and a no-treatment control. We compared the speed at which these treatments acted, by measuring the percentage of sand flies killed both immediately after exposure to the treatment for 1 hour, as well as the number that had died 24 h after the 1 hour exposure. We repeated the experiment at 6 and 12 months following application to test the effectiveness of each treatment over time.

**Results:**

When first applied, the lambda-cyhalothrin killed more sand flies in the first hour than the permethrin-impregnated netting. However, the effectiveness of the lambda-cyhalothrin diminished over time, so that there was no difference between the two treatments at 12 months. Both killed more sand flies than the control. When measured 24 h following exposure, both test treatments had killed close to 100% of sand flies when first applied, but while the lethal effect of the netting was maintained at close to 100% over 12 months, the effectiveness of the residual insecticide diminished to approximately 80% after 6 months.

**Conclusions:**

The results of these initial laboratory experiments indicate that covering surfaces with insecticide impregnated netting material may provide a longer-lasting solution for killing sand flies than residual spraying. Field trials are needed to identify the feasibility of treating surfaces with netting or similar impregnated materials as part of a control program. In targeting *L. longipalpis*, the greatest benefits may be seen in treating animal sheds with netting, where these sand flies aggregate in large numbers, and which can be difficult to treat repeatedly by conventional spraying.

## Background

Leishmaniasis remains a serious neglected disease, with more than 350 million people at potential risk worldwide, and an estimated 50 000 deaths per year [1]. Infection by the etiologic agents, protozoan parasites of the genus *Leishmania* (Kinetoplastida: Trypanosomatidiae)*,* can lead to severe disfigurement (cutaneous and mucocutaneous leishmaniasis), and can be fatal if left untreated (visceral leishmaniasis). As there is currently no effective human vaccine [2], strategies that aim to control leishmaniasis most often target infected animal reservoirs, through culling (which is unpopular and often ineffective [3]), or populations of the sand fly vectors (Diptera: Psychodidae), which transmit the disease [4-6]. Approximately 70 species of sand fly have been identified as vectors of leishmaniasis [7], and although ecology and feeding behaviour vary between species, transmission always occurs through blood-feeding by female sand flies on an infected animal or human host.

Sand fly control programmes often rely on spraying potential resting sites (for example, animal or human houses) with residual insecticides [5]. While regular spraying can offer some protection to human populations from infection [8], such strategies are often difficult to sustain effectively, particularly in rural communities, where there may be large numbers of potential resting sites that must be visited and resprayed on a regular basis (for example, once every six months in endemic areas [9]). Spraying also requires training to be conducted effectively, in order to ensure that the correct concentration of insecticide is applied to kill sand flies, while minimizing exposure to sub-lethal amounts which might promote the onset of resistance [10,11]. In Brazil, where over 90% of visceral leishmaniasis cases in South America occur, insecticide is applied only after a human case has been identified because of the logistics associated with spraying [12].

A longer-lasting solution for killing sand flies, which does need to be reapplied with the same frequency as residual spraying, could dramatically reduce the cost of leishmaniasis control programmes, facilitating their adoption by disease control agencies. Insecticide-treated bed nets (ITNs) offer a cost-effective alternative to residual spraying, offering personal protection from nuisance insects [13], mosquito vectors of malaria [14] and sand fly vectors of cutaneous leishmaniasis [15,16]. In Brazil, trials of deltamethrin-impregnated bed nets have demonstrated important entomological effects associated with their use, reducing the landing rate of the American visceral leishmaniasis (AVL) vector *Lutomyia longipalpis* on humans, and increasing sand fly mortality, compared to untreated nets [15]. However, as a consequence of the crepuscular feeding activity of *L. longipalpis*, nets used in this way in practice offer limited protection against AVL as more than 50% of bites occur in the early evening, before householders are sleeping under nets [17].

An alternative to using insecticide-releasing materials such as bednets could be to apply the netting to surfaces where sand flies rest, as a direct alternative to residual spraying. Modern long-lasting insecticide netting (LLIN) can withstand washing and will release insecticide for a number of years (e.g. up to 5 for some formulations), without the need for retreatment [18]. If suitably effective at killing *L. longipalpis*, deployment of such nets as a means of delivering insecticide, at lethal dosage levels without the need for complex training and validation of dosage levels, at sand fly resting sites could be more cost-effective than repeated spraying with residual insecticides. However, while LLINs are now recommended for use in malaria vector control programmes [18] their effectiveness in killing *L. longipalpis*, and their longevity compared to conventional insecticides for *L. longipalpis* control, has not been ascertained under controlled conditions.

The aim of this study was to determine whether LLIN could be used as an effective direct replacement for residual insecticide spraying as a means of killing *L. longipalpis*. The lethal effect of permethrin-impregnated netting against female *L. longipalpis* was compared with that of a micro-encapsulated formulation of lambda-cyhalothrin, an insecticide recommended for use for *L. longipalpis* control in Brazil [12], designed to provide long-lasting residual control of insect pests [19]. Our goal was not to compare directly between the two chemical insecticides used in the LLIN and the micro-encapsulated spray, which are present at different concentrations, but rather to determine which of these two products, already marketed for use in protecting against blood-feeding insects, would provide a longer-lasting solution for killing *Lutzomyia longipalpis* at sand fly resting sites.

To assess the relative longevity of these two insecticide delivery methods, we compared their effectiveness both shortly after their application to a wooden surface, and again after periods of six and twelve months. To test for differences in the speed at which these insecticide treatments act, their lethal effect was measured on a group of sand flies after they had been exposed to a treated surface for 1 hour (immediate effect) and again on the same group of sand flies after a period of 24 h had elapsed since their initial exposure (delayed effect).

## Methods

### Surface treatment

Insecticides were applied to 80 mm × 80 mm sections of 10 mm-thick marine plywood (Homebase Limited, Buckinghamshire, UK). For netting treatments, a single layer of blue Olyset® (Sumitomo Chemical Co. Ltd., Tokyo, Japan) netting, consisting of woven polyester hexagons (3 mm approximate diameter) impregnated with 2% permethrin, was secured over the entire surface using drawing pins. For residual insecticide treatment, surfaces were treated with a 20 mg a.i.m^-2^ solution of microencapsulated lambda-cyhalothrin (Demand CS®; BASF PLC, Cheshire, UK), using a paintbrush and allowed to dry for 24 h. This insecticide formulation was chosen as previous studies have demonstrated its relatively long-lasting efficacy against sand flies compared to non-microencapsulated formulations of other insecticides [8], and a similar formulation has been shown to kill *L. longipalpis* in the field at this concentration [20,21]. The concentration applied is that recommended by the manufacturer for use against mosquitoes, and is not dissimilar to that endorsed by the Brazilian health authorities for sand fly control (30 mg a.i.m^-2^, [12]). This product therefore most likely represents the strongest possible competitor for comparison against insecticide impregnated materials for long-lasting control of sand flies. A third category of surfaces were left untreated as controls.

### Insecticide testing

For each replicate, the open face (80 mm diameter) of a 118 ml polystyrene cup (Dart Products Limited, West Midlands, UK) was attached to the treated plywood surface using adhesive tape. Twenty-five 6 day-old female *L. longipalpis* from a laboratory colony [22], were then introduced via a small hole cut in the base of the cup. The hole was sealed using a cotton wool plug, and the treated surface held vertically for 1 h, after which time the cup was removed in a Nylon cage (18 cm × 18 cm × 18 cm), and the number of sand flies alive and dead counted. The cage containing the remaining sand flies was kept within a humidified polythene bag for 24 h at 27°C, when the numbers of dead and alive flies were again recorded. The period of exposure used was chosen to allow comparison with a previous study that used the same time period in similar bioassays measuring the long term effectiveness of lamba-cyhalothrin against sand flies when applied to internal and external surfaces in the field [8].

Ten replicates were performed for each treatment (netting, insecticide and untreated control), using different sand flies and treated surfaces for each replicate. To assess the relative longevity of each treatment, the same testing procedure was repeated after six and twelve months, with treated surfaces kept free-standing under dry indoor conditions in the UK until they were retested.

### Data analysis

The percentage of sand flies killed by the three treatments over time was compared by ANOVA. Firstly, percentage of sand flies killed immediately following 1 h of exposure (number of sand flies killed/number exposed * 100) was entered as the dependent variable, with treatment (control, netting and insecticide) and time since application of treatment (0 months, 6 months and 12 months) entered as factors. An interaction term (treatment by time since treatment) was also included, to test for a potential difference in the way in which the effectiveness of the three treatments changed over time. Where significant effects were found in the two-way ANOVA, an individual one-way ANOVA was used to examine how the effectiveness of each treatment changed with time, with time points compared through Tukey’s post-hoc tests. Similarly, one-way ANOVA and Tukey’s post-hoc tests were used to clarify differences in the effectiveness of each treatment at each time point.

The analysis was then repeated with percentage of sand flies killed after 24 h entered as the dependent variable, to test for differences between the three treatments over time in their effectiveness in killing sand flies over a more prolonged period following exposure. All analyses were performed in R version 2.15.2 [23], and for both full models residuals were approximately normally distributed, indicating an appropriate fit to the data set.

## Results

### Immediate efficacy of insecticide treatments

Overall, a significant difference was found between the three treatments (control, netting and insecticide) in their effectiveness in killing sand flies within 1 h following exposure (immediate effect) (F_2, 81_ = 107.6, P <0.001) and the immediate lethal effect of treatment was also found to change over time (F_2, 81_ = 53.1, P < 0.001). The presence of a significant interaction term (treatment by time since treatment; F_4, 81_ = 49.4, P <0.001) indicated that, overall, the three treatments differed in how their effectiveness changed over time.

**Figure 1:**
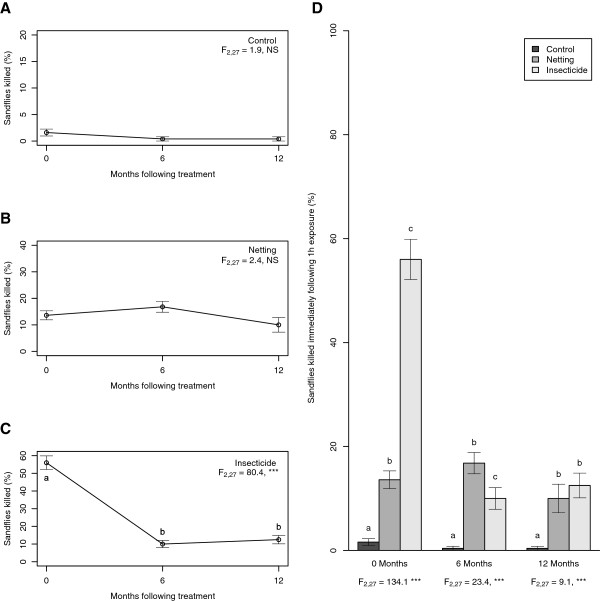
**Immediate efficacy of insecticide treatments.** Percentage of sand flies killed immediately following 1 h exposure to plywood surfaces, untreated (control), covered with permethrin-impregnated netting or treated with lambda-cyhalothrin (insecticide). **A-C**: change in effectiveness of each treatment over time. F-values refer to ANOVA across all three time points, different letters indicate significant differences between time points (Tukey’s post-hoc test). **D**: comparison of effectiveness of each treatment at each time point. F-values refer to ANOVA across all three categories at each time point; different letters indicate significant differences between categories at each time point. * = P < 0.05, ** = P < 0.01, *** = P < 0.05. NS = Not Significant.

Subsequent analysis revealed that the immediate lethal effect of the control (Figure 1A) and netting treatments (Figure 1B) remained constant, killing on average (mean ± SEM) 0.8% ± 0.3 and 13.5% ±1.3 of exposed sand flies respectively. The effectiveness of the insecticide treatment, however, dropped significantly over time, from 56% ± 3.9 immediately after application to 10.0% ± 2.1 at 6 months, whereafter its effectiveness remained relatively constant up to 12 months (Figure 1C).

The residual insecticide killed more sand flies at 0 months compared to the netting (Figure 1D). However, as the immediate lethal effect of the insecticide decreased over time, the netting became relatively more effective so that by 12 months, there was no significant difference between the two. Throughout, both micro-encapsulated insecticide and netting treatments killed significantly more sand flies than the no-treatment control (Figure 1D).

### Efficacy of insecticide treatments over 24 h

The three treatments differed overall in their lethal effect on sand flies in the 24 h period following exposure (F_2, 81_ = 1026, P <0.001), and overall their effectiveness was found to change over time since application. As above, the extent to which the effectiveness of each treatment diminished was also found to differ (F_2, 81_ = 6.6, P <0.001).

**Figure 2:**
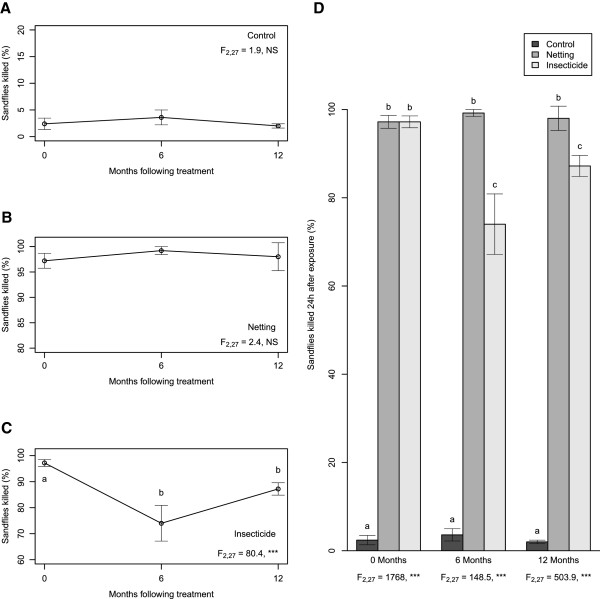
**Efficacy of insecticide treatments over 24 h.** Percentage of sand flies killed within 24 h following 1 h exposure to plywood surfaces, untreated (control), covered with permethrin-impregnated netting or treated with lambda-cyhalothrin (insecticide). **A-C**: change in effectiveness of each treatment over time. F-values refer to ANOVA across all three time points, different letters indicate significant differences between time points (Tukey’s post-hoc test). **D**: comparison of effectiveness of each treatment at each time point. F-values refer to ANOVA across all three categories at each time point; different letters indicate significant differences between categories at each time point. * = P < 0.05, ** = P < 0.01, *** = P < 0.05. NS = Not Significant.

In subsequent analyses, the lethal effects of the netting and control treatments were not found to differ over the 12 months following application (Figure 2A, B). The netting killed 98.1% ± 0.6 of sand flies exposed, compared to the control, which killed 2.7% ± 0.6 sand flies exposed within 24 h. The effectiveness of the micro-encapsulated insecticide, however, dropped from 97.2% ± 1.3 immediately after treatment, to 74.0% ± 6.9 and 87.2% ± 2.4 six and twelve months after application respectively (Figure 2C).

In comparing the three treatments at each time point, both the netting and insecticide killed close to 100% of sand flies exposed within 24 h when first applied, with no difference between the effectiveness of the two treatments (Figure 2D). However, at 6 and 12 months, the netting killed significantly more sand flies than the insecticide. Both treatments killed significantly more sand flies than the control throughout the 12 months of the experiment (Figure 2D).

## Discussion

Long-lasting insecticide impregnated netting has not previously been tested as a mechanism for delivering insecticide to kill *L. longipalpis*. In this laboratory study, Olyset® netting attached to a wooden surface killed as many sand flies as lambda-cyhalothrin within 24 h when first applied, and might therefore be considered as a feasible alternative to residual spraying.

Lambda-cyhalothrin has been shown to be effective in killing sand flies under experimental conditions [8,10,20,21] and is recommended for use by the Brazilian Ministry of Health for leishmaniasis control [12]. It was chosen for comparison in this study because the micro-encapsulated formulation is designed to provide a relatively long-lasting residual effect compared to conventional insecticide formulations [19]. Here, efficacy of the lambda-cyhalothrin treatment declined to 74% over 6 months: a reduction similar to that observed against the cutaneous leishmaniasis vector *Lutzomyia verrucarum* when sprayed on outside walls in Peru [8]. In comparison, the permethrin-impregnated netting maintained its effectiveness at close to 100% lethality 24 h post exposure for the twelve months of the study. Olyset netting might, therefore, provide a longer-lasting solution for killing *L. longipalpis*: however, permethrin is not currently used by the Brazilian Ministry of Health for sand fly control [12], and while its efficacy under laboratory conditions is shown here, trials performed using a variety of indoor and outdoor surfaces in Brazil will be needed to confirm its effectiveness in the field. Alternatively, trials could be conducted using long-lasting netting impregnated with deltamethrin [24], an insecticide that is already approved for sand fly control by the Brazilian Government [12].

While both residual insecticide and netting treatments killed close to 100% of *L. longipalpis* within 24 h when first applied, the immediate mortality-inducing effect of the netting was significantly weaker than that of the lambda-cyhalothrin, with fewer flies killed immediately following 1 h of exposure. A trade-off may therefore exist between the faster immediate efficacy of the residual insecticide when first applied, and the overall longevity of the netting. The speed at which an insecticide intervention acts may be most important when used for personal protection: for example, as a bed net or bedroom wall covering when flies are in close proximity to the target host, and must be killed before they attempt to blood-feed.

Erecting insecticide impregnated materials as an alternative to residual spraying (for example as curtains) might reduce the number of *L. longipalpis* resting on walls, and therefore biting rate on humans, for a longer period. It may also be a more acceptable solution to householders concerned with the potential health risks of spraying insecticides indoors in close proximity to humans and domestic animals. Impregnated netting used in this way has been shown to be effective against several endophilic species of sand fly, which readily enter houses in search of a blood meal [25-29]. However, the usefulness of such a strategy against vectors that are more abundant in the peridomestic environment, such as *L. longipalpis*[30], is unclear [31]. In this instance, greater reductions in local vector populations could more easily be achieved by placing the netting in animal houses where sand flies congregate in the early evening [32]. However, previous trials in Brazil have demonstrated that insecticide-treated cotton sheets (‘targets’) sprayed with lambda-cyhalothrin were relatively poor in controlling *L. longipalpis* in chicken sheds compared with residual spraying [21]. A relatively large proportion of the internal surface of the animal house might, therefore, have to be treated with the netting for such a strategy to have a significant impact on sand fly numbers in the proximity of human houses, and thereby reduce biting risk and subsequent transmission. Attaching the netting to the internal walls of animal houses using staples or nails, while leaving openings free for domestic animals to enter and exit, may provide the most acceptable means of application for householders. However, the efficacy of this solution in killing *L. longipalpis* will depend on the availability of solid internal surfaces on which sand flies can alight. Furthermore, sand flies may not remain on insecticide treated surfaces for as long as the 1 h exposure period used in this study, instead being diverted away or immobilized once they come into close proximity with a treated surface [17]. Addition of an attractive bait may be needed to prevent sand flies being displaced to non-treated sites, potentially closer to human habitation [20,21].

Whether or not insecticide-impregnated materials would be adopted by disease control agencies for use against *L. longipalpis* would depend in part on their cost compared to insecticides, and the logistics of their implementation and maintenance. Although netting may be more bulky to transport, its deployment would not require either the specialist safety equipment or training needed for insecticide spraying, and it would not have to be replaced as frequently. Further efficiency savings could be made if a suitable insecticide impregnated material could be deployed to protect against mosquito vectors of dengue, and malaria, as well as leishmaniasis-transmitting sand flies. However, while insecticide impregnated linings have been tested against mosquitoes, it appears they are most effective when applied in combination with bed nets or residual spraying [33-36]. They may therefore, be most useful in offering protection against exophilic, exophagic or zoophilic sand flies, where bednets offer little protection against crepuscular feeding, and residual spraying of indoor and outdoor resting sites cannot be maintained effectively.

## Conclusion

The results of this laboratory study indicate that LLINs could provide a longer-lasting alternative for controlling numbers of *L. longipalpis* in animal houses, compared to even the most durable of sprayed-on residual insecticide treatments. Field studies are needed to determine how these nets could most effectively be applied in chicken sheds and other animal shelters, where sand flies congregate in large numbers.

## Competing interests

The authors declare that they have no competing interests.

## Authors’ contributions

DPB and JGH conceived the study. DPB carried out the study, performed the analysis and drafted the manuscript. Both authors read and approved the final manuscript.
